# l-Histidinium dipicrate dihydrate

**DOI:** 10.1107/S1600536813013949

**Published:** 2013-05-25

**Authors:** M. Sethuram, M. V. Rajasekharan, M. Dhandapani, G. Amirthaganesan, M. NizamMohideen

**Affiliations:** a Department of Chemistry, Sri Ramakrishna Mission Vidyalaya College of Arts and Science, Coimbatore 641 020, Tamil Nadu, India; b School of Chemistry, University of Hyderabad, Hyderabad 500 046, Andhra Pradesh, India; c Department of Physics, The New College (Autonomous), Chennai 600 014, India

## Abstract

In the title mol­ecular salt, C_6_H_11_N_3_O_2_^2+^·2C_6_H_2_N_3_O_7_^−^·2H_2_O, the histidine mol­ecule exists as a histidinium dication, being protonated at the N atom of the imidazole ring. The charges are balanced by two picrate anions and the compound crystallizes as a dihydrate. In the crystal, the components are linked *via* N—H⋯O and O—H⋯O hydrogen bonds and weak C—H⋯O inter­actions, forming a three-dimensional supermolecular structure.

## Related literature

For the role of hydrogen bonding in the construction of supra­molecular structures, see: Braga *et al.* (2004 ▶); Harrowfield *et al.* (1995 ▶). For picrates of biologically important mol­ecules, see: Harrison *et al.* (2007 ▶); Swamy *et al.* (2007 ▶); Bibal *et al.* (2003 ▶); Olsher *et al.* (1996 ▶). For bond angles in related structures, see: Yang *et al.* (2001 ▶).
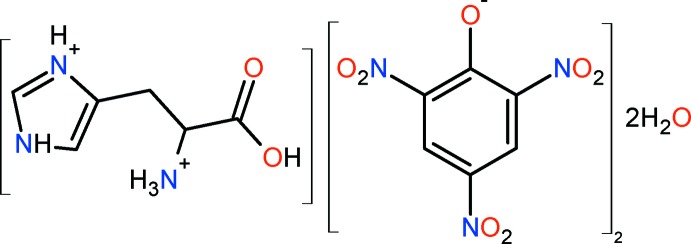


## Experimental

### 

#### Crystal data

C_6_H_11_N_3_O_2_^2+^·2C_6_H_2_N_3_O_7_^−^·2H_2_O
*M* *_r_* = 649.42
Monoclinic, 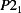

*a* = 6.6060 (4) Å
*b* = 25.7003 (13) Å
*c* = 7.9627 (5) Å
β = 107.532 (7)°
*V* = 1289.08 (13) Å^3^
*Z* = 2
Mo *K*α radiation
μ = 0.15 mm^−1^
*T* = 293 K
0.20 × 0.15 × 0.10 mm

#### Data collection

Bruker Kappa APEXII CCD diffractometer
Absorption correction: multi-scan (*SADABS*; Sheldrick, 2004 ▶) *T*_min_ = 0.970, *T*_max_ = 0.985
5817 measured reflections
2982 independent reflections
2560 reflections with *I* > 2σ(*I*)
*R*_int_ = 0.020

#### Refinement

*R*[*F* ^2^ > 2σ(*F* ^2^)] = 0.039
*wR*(*F* ^2^) = 0.088
*S* = 1.09
2982 reflections
439 parameters
7 restraints
H atoms treated by a mixture of independent and constrained refinement
Δρ_max_ = 0.21 e Å^−3^
Δρ_min_ = −0.18 e Å^−3^

### 

Data collection: *APEX2* (Bruker, 2004 ▶); cell refinement: *APEX2* and *SAINT* (Bruker, 2004 ▶); data reduction: *SAINT* and *XPREP* (Bruker, 2004 ▶); program(s) used to solve structure: *SHELXS97* (Sheldrick, 2008 ▶); program(s) used to refine structure: *SHELXL97* (Sheldrick, 2008 ▶); molecular graphics: *ORTEP-3 for Windows* (Farrugia, 2012 ▶) and *Mercury* (Macrae *et al.*, 2008 ▶); software used to prepare material for publication: *WinGX* (Farrugia, 2012 ▶) and *PLATON* (Spek, 2009 ▶).

## Supplementary Material

Crystal structure: contains datablock(s) global, I. DOI: 10.1107/S1600536813013949/su2602sup1.cif

Structure factors: contains datablock(s) I. DOI: 10.1107/S1600536813013949/su2602Isup2.hkl

Supplementary material file. DOI: 10.1107/S1600536813013949/su2602Isup3.cml

Additional supplementary materials:  crystallographic information; 3D view; checkCIF report

## Figures and Tables

**Table 1:** Hydrogen-bond geometry (Å, °)

*D*—H⋯*A*	*D*—H	H⋯*A*	*D*⋯*A*	*D*—H⋯*A*
N1—H1*A*⋯O10	0.89	2.19	2.909 (3)	138
N1—H1*A*⋯O12	0.89	2.11	2.841 (4)	139
N1—H1*B*⋯O18*W*	0.89	1.85	2.700 (5)	158
N1—H1*C*⋯O15^i^	0.89	2.16	3.007 (4)	159
N2—H2*B*⋯O10	0.91 (5)	1.86 (5)	2.709 (4)	154 (4)
N2—H2*B*⋯O16	0.91 (5)	2.49 (4)	3.125 (4)	128 (3)
N3—H3⋯O9^ii^	0.86	2.56	2.992 (5)	112
N3—H3⋯O14^ii^	0.86	2.25	3.077 (4)	160
O2—H2⋯O3^iii^	0.82	1.86	2.657 (3)	165
O17*W*—H17*A*⋯O5^iv^	0.84 (2)	2.27 (3)	3.082 (4)	162 (9)
O17*W*—H17*B*⋯O3	0.84 (2)	2.14 (8)	2.864 (4)	144 (12)
O18*W*—H18*A*⋯O17*W* ^v^	0.83 (2)	1.83 (2)	2.664 (5)	176 (5)
O18*W*—H18*B*⋯O7	0.83 (2)	2.32 (4)	3.005 (5)	140 (6)
C3—H3*B*⋯O10	0.97	2.59	3.210 (4)	122
C9—H9⋯O8^vi^	0.93	2.40	3.177 (4)	141
C17—H17⋯O11^iv^	0.93	2.36	3.177 (3)	147

Symmetry codes: (i) 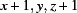; (ii) 
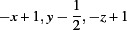
; (iii) 
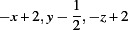
; (iv) 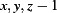; (v) 
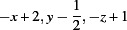
; (vi) 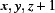.

**Fig. 1.**
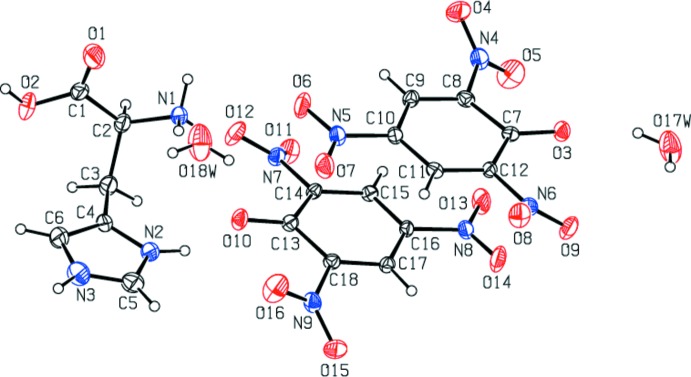
View of the molecular structure of the title compound, showing the atom labelling. Displacement ellipsoids are drawn at the 30% probability level.

**Fig. 2.**
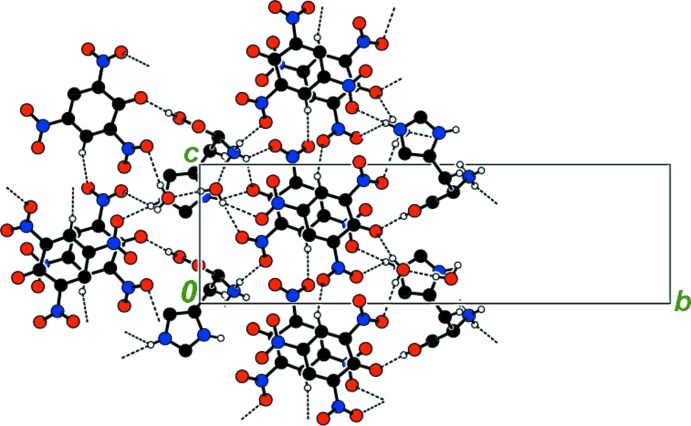
A partial view along the a axisof the crystal packing of the title compound. Dashed lines indicate N—H···O and O—H···O hydrogen bonds and weak C—H···O interactions (see Table 1 for details).

## supplementary crystallographic information

### Comment

Intermolecular and inter-ionic hydrogen bonding interactions, which are not only the strongest of the noncovalent interactions but also highly directional, play an important role in constructing supramolecular structures (Braga *et al.*, 2004). Picrate is generally used as an accompanying ion in many systems involving extraction and transport of metal ions to improve the extractability (Bibal *et al.*, 2003). Picrate interacts as a monodentate, bidentate and tridentate ligand (Olsher *et al.*, 1996). Furthermore, picrate is a penta-dentate ligand when it coordinates with cation by chelating pairs of oxygen atoms from *p*-nitro groups of adjacent picrates, and with successive cations linking the array into a two or three-dimensional network (Harrowfield *et al.*, 1995) and picrates of biologically important molecules (Harrison *et al.*, 2007; Swamy *et al.*, 2007). We have prepared a new picrate of *L*-Histidinium hydrate and its crystal structure is reported herein.

The asymmetric unit of the title compound, Fig. 1, contains an *L*-histidinium cation, two picrate anions and two water molecules. The histidine molecule exists as an histidinium ion due to the protonation at the N atom of the imidazole ring. The charges are equilibrated by two picrate anions and crystallizes as a dihydrate. The imidazole ring (N2/N3/C4/C5/C6) makes a dihedral angle of 5.0 (2) and 4.9 (2)° with the benzene rings (C7—C12 and C13—C18), respectively, of the picrate anions.

The picrate anions adopt the keto form with C7—O3 and C13—O10 bond distance of 1.261 (4) and 1.249 (4) Å, C7—C8, C7—C12, C13—C14 and C13—C18 bond distance of 1.445 (4), 1.444 (4), 1.451 (4) and 1.450 (4) Å, respectively, which is longer than the other C—C bond lengths (between 1.374 (5) to 1.460 (4) Å) in the benzene ring. The bond angles C12—C7—C8 and C14—C13—C18 is 111.3 (3) and 110.6 (3)°, respectively, which is the case in some picrate complexes, while the corresponding bond angle of picric acid is 116.4 (5)° [Yang *et al.*, 2001]. In the picrate anion the depronated phenolate oxygen atom deviates slightly from the plane of the benzene ring (torsion angle O3-C7-C8-C9 = -175.4 (3) ° and O10-C13-C14-C15 = 178.2 (3)°). The twist angles between the benzene rings (C7—C12 and C13—C18) and the *ortho* nitro groups (N4, N6, N7 and N9) are 41.5 (2), 32.4 (2), 32.2 (2) and 34.8 (2), respectively. The *para*-positioned nitro groups are twisted by 3.4 (2)° (N5) and 5.8 (2)° (N8), and are most likely influenced by a weak hydrogen bond interaction (O18—H18B···O7). The picrate ions are stacked head-to-tail, presumably as a result of charge-transfer interactions.

In the crystal the cation, the picrate anions and the water molecules of crystallization are involved in N—H···O and O—H···O hydrogen bonds and week C—H···O interactions, to form a three-dimensional supramolecular network (Table 1 and Fig. 2).

### Experimental

1:2 stoichiometric proportions of analar grades *L*-histidine and picric acid (E-Merck) were dissolved in a triply distilled water and ethanol mixture and the two solutions were thoroughly mixed together using mechanical stirrer for about three hours. The clear yellow solution obtained was filtered off to get the crude material. The material was re-dissolved in a water-ethanol solvent mixture and kept aside without any mechanical movement for crystal growth in a dust free environment. Bright yellowish crystals that formed in 5 days were collected carefully from the mother liquor. Several re-crystallizations were done to get ultra pure crystals. The yield in the reaction was ca. 60%. Analysis calc. for C_18_H_18_N_9_O_18 _: C: 33.34, H: 2.79, N: 19.44 %; Found: C: 33.21, H: 3.74, N: 19.60%.

### Refinement

The water molecule H-atoms, the methine (CH) H atom, and the CH and one NH H atom of the imidazole ring, were located in a difference Fourier map and freely refined. The OH, the NH_3, _one NH H atom of the imidazole ring, and the CH~2~ H atoms were positioned geometrically and refined using a riding model: O-H = 0.82 Å, N—H = 0.89 Å (NH_3_), N—H = 0.86 Å, C—H = 0.97 Å for CH_2_ H atoms, with *U*_iso_(H) = 1.5U_eq_(O,N) for the OH and NH_3_ H atoms and = 1.2U_eq_(N,C) for other H atoms.

### Crystal data

**Table:**

C_6_H_11_N_3_O_2_^2+^·2C_6_H_2_N_3_O_7_^−^·2H_2_O	*F*(000) = 668
*M**_r_* = 649.42	*D*_x_ = 1.673 Mg m^−^^3^
Monoclinic, *P*2_1_	Mo *K*α radiation, λ = 0.71073 Å
Hall symbol: P 2yb	Cell parameters from 5817 reflections
*a* = 6.6060 (4) Å	θ = 2.4–31.1°
*b* = 25.7003 (13) Å	µ = 0.15 mm^−^^1^
*c* = 7.9627 (5) Å	*T* = 293 K
β = 107.532 (7)°	Square, yellow
*V* = 1289.08 (13) Å^3^	0.20 × 0.15 × 0.10 mm
*Z* = 2	

### Data collection

**Table:**

Bruker Kappa APEXII CCD diffractometer	2982 independent reflections
Radiation source: fine-focus sealed tube	2560 reflections with *I* > 2σ(*I*)
Graphite monochromator	*R*_int_ = 0.020
ω and φ scans	θ_max_ = 28.9°, θ_min_ = 2.8°
Absorption correction: multi-scan (*SADABS*; Sheldrick, 2004)	*h* = −8→8
*T*_min_ = 0.970, *T*_max_ = 0.985	*k* = −26→34
5817 measured reflections	*l* = −10→9

### Refinement

**Table:**

Refinement on *F*^2^	Secondary atom site location: difference Fourier map
Least-squares matrix: full	Hydrogen site location: inferred from neighbouring sites
*R*[*F*^2^ > 2σ(*F*^2^)] = 0.039	H atoms treated by a mixture of independent and constrained refinement
*wR*(*F*^2^) = 0.088	*w* = 1/[σ^2^(*F*_o_^2^) + (0.0341*P*)^2^ + 0.2411*P*] where *P* = (*F*_o_^2^ + 2*F*_c_^2^)/3
*S* = 1.09	(Δ/σ)_max_ < 0.001
2982 reflections	Δρ_max_ = 0.21 e Å^−^^3^
439 parameters	Δρ_min_ = −0.18 e Å^−^^3^
7 restraints	Extinction correction: *SHELXL97* (Sheldrick, 2008), Fc^*^=kFc[1+0.001xFc^2^λ^3^/sin(2θ)]^-1/4^
Primary atom site location: structure-invariant direct methods	Extinction coefficient: 0.0095 (12)

### Special details

**Table:**

Geometry. All e.s.d.'s (except the e.s.d. in the dihedral angle between two l.s. planes) are estimated using the full covariance matrix. The cell e.s.d.'s are taken into account individually in the estimation of e.s.d.'s in distances, angles and torsion angles; correlations between e.s.d.'s in cell parameters are only used when they are defined by crystal symmetry. An approximate (isotropic) treatment of cell e.s.d.'s is used for estimating e.s.d.'s involving l.s. planes.
Refinement. Refinement of *F*^2^ against ALL reflections. The weighted *R*-factor *wR* and goodness of fit *S* are based on *F*^2^, conventional *R*-factors *R* are based on *F*, with *F* set to zero for negative *F*^2^. The threshold expression of *F*^2^ > σ(*F*^2^) is used only for calculating *R*-factors(gt) *etc*. and is not relevant to the choice of reflections for refinement. *R*-factors based on *F*^2^ are statistically about twice as large as those based on *F*, and *R*- factors based on ALL data will be even larger.

### Fractional atomic coordinates and isotropic or equivalent isotropic displacement parameters (Å^2^)

**Table:**

	*x*	*y*	*z*	*U*_iso_*/*U*_eq_	
O1	1.1170 (5)	−0.00275 (12)	1.2736 (5)	0.0774 (11)	
O2	0.8469 (5)	−0.04330 (11)	1.3342 (4)	0.0566 (8)	
H2	0.9346	−0.0664	1.3719	0.085*	
O3	0.9237 (4)	0.37442 (9)	0.5194 (3)	0.0366 (6)	
O4	1.1711 (5)	0.33930 (12)	1.0182 (3)	0.0618 (9)	
O5	0.9113 (6)	0.38681 (13)	0.8677 (4)	0.0659 (9)	
O6	1.0105 (5)	0.15561 (11)	0.8787 (3)	0.0527 (7)	
O7	0.9511 (5)	0.13208 (10)	0.6075 (3)	0.0507 (7)	
O8	0.9119 (4)	0.26246 (10)	0.1693 (3)	0.0456 (6)	
O9	0.7415 (5)	0.33288 (10)	0.1914 (3)	0.0489 (7)	
O10	0.5060 (4)	0.11319 (9)	0.8159 (3)	0.0379 (6)	
O11	0.4555 (4)	0.23048 (10)	1.1305 (3)	0.0443 (6)	
O12	0.6326 (5)	0.16004 (10)	1.1346 (3)	0.0505 (7)	
O13	0.4413 (5)	0.35254 (9)	0.6731 (4)	0.0529 (7)	
O14	0.3963 (5)	0.32377 (10)	0.4098 (3)	0.0522 (7)	
O15	0.2242 (5)	0.14022 (12)	0.3056 (3)	0.0565 (8)	
O16	0.4623 (6)	0.09005 (11)	0.4743 (4)	0.0668 (9)	
N1	0.8717 (5)	0.07237 (10)	1.0875 (4)	0.0354 (6)	
H1A	0.7843	0.0987	1.0431	0.053*	
H1B	0.8912	0.0535	1.0000	0.053*	
H1C	0.9959	0.0848	1.1535	0.053*	
N2	0.4952 (5)	0.00857 (12)	0.7727 (4)	0.0380 (7)	
N3	0.4987 (6)	−0.07402 (13)	0.7471 (5)	0.0590 (10)	
H3	0.4956	−0.1045	0.7020	0.071*	
N4	1.0229 (5)	0.34810 (12)	0.8865 (4)	0.0388 (7)	
N5	0.9722 (4)	0.16635 (11)	0.7214 (4)	0.0353 (7)	
N6	0.8457 (5)	0.29442 (11)	0.2552 (3)	0.0322 (6)	
N7	0.5317 (4)	0.19734 (11)	1.0568 (3)	0.0312 (6)	
N8	0.4235 (4)	0.31696 (11)	0.5679 (4)	0.0315 (6)	
N9	0.3649 (5)	0.13122 (11)	0.4434 (3)	0.0364 (7)	
C1	0.9350 (6)	−0.00520 (14)	1.2718 (5)	0.0423 (9)	
C2	0.7771 (6)	0.03902 (13)	1.1981 (4)	0.0344 (7)	
C3	0.5503 (5)	0.02145 (14)	1.0983 (4)	0.0365 (8)	
H3A	0.4943	0.0022	1.1793	0.044*	
H3B	0.4629	0.0522	1.0624	0.044*	
C4	0.5280 (5)	−0.01146 (13)	0.9395 (4)	0.0331 (7)	
C5	0.4757 (6)	−0.03046 (16)	0.6596 (5)	0.0493 (10)	
C6	0.5283 (7)	−0.06396 (16)	0.9212 (5)	0.0504 (10)	
C7	0.9258 (5)	0.32737 (12)	0.5650 (4)	0.0256 (6)	
C8	0.9775 (5)	0.30948 (13)	0.7450 (4)	0.0283 (7)	
C9	0.9939 (5)	0.25863 (13)	0.7963 (4)	0.0275 (7)	
H9	1.0308	0.2500	0.9151	0.033*	
C10	0.9549 (5)	0.22013 (12)	0.6693 (4)	0.0265 (6)	
C11	0.9070 (4)	0.23282 (12)	0.4924 (4)	0.0259 (6)	
H11	0.8856	0.2068	0.4077	0.031*	
C12	0.8915 (5)	0.28421 (11)	0.4435 (4)	0.0235 (6)	
C13	0.4825 (5)	0.15872 (12)	0.7585 (4)	0.0260 (6)	
C14	0.5012 (5)	0.20440 (12)	0.8692 (4)	0.0232 (6)	
C15	0.4816 (5)	0.25485 (12)	0.8099 (4)	0.0246 (6)	
H15	0.4977	0.2825	0.8884	0.030*	
C16	0.4375 (5)	0.26384 (12)	0.6310 (4)	0.0237 (6)	
C17	0.3974 (4)	0.22294 (12)	0.5116 (4)	0.0247 (6)	
H17	0.3562	0.2293	0.3910	0.030*	
C18	0.4195 (5)	0.17329 (12)	0.5740 (4)	0.0249 (6)	
O17W	1.0107 (7)	0.43238 (13)	0.2418 (5)	0.0724 (10)	
O18W	1.0051 (8)	0.03423 (14)	0.8230 (5)	0.0779 (11)	
H17A	1.006 (14)	0.415 (3)	0.150 (7)	0.18 (4)*	
H17B	1.04 (2)	0.411 (3)	0.325 (9)	0.31 (7)*	
H18A	1.001 (9)	0.0022 (8)	0.808 (7)	0.086 (19)*	
H18B	0.977 (15)	0.049 (2)	0.726 (5)	0.19 (4)*	
H2A	0.763 (6)	0.0623 (16)	1.296 (5)	0.039 (10)*	
H6	0.539 (7)	−0.0916 (19)	0.999 (6)	0.064 (13)*	
H5	0.455 (7)	−0.029 (2)	0.536 (6)	0.068 (13)*	
H2B	0.479 (7)	0.0432 (19)	0.751 (5)	0.053 (12)*	

### Atomic displacement parameters (Å^2^)

**Table:**

	*U*^11^	*U*^22^	*U*^33^	*U*^12^	*U*^13^	*U*^23^
O1	0.0441 (17)	0.047 (2)	0.123 (3)	0.0016 (14)	−0.0029 (17)	0.0338 (19)
O2	0.0723 (19)	0.0389 (16)	0.0567 (16)	0.0113 (15)	0.0166 (14)	0.0245 (13)
O3	0.0544 (15)	0.0211 (12)	0.0300 (11)	−0.0025 (11)	0.0064 (10)	−0.0010 (9)
O4	0.083 (2)	0.0564 (19)	0.0295 (13)	−0.0121 (17)	−0.0084 (13)	−0.0061 (13)
O5	0.096 (2)	0.0489 (19)	0.0517 (16)	0.0160 (18)	0.0213 (15)	−0.0173 (14)
O6	0.0750 (19)	0.0426 (16)	0.0386 (14)	−0.0024 (14)	0.0143 (13)	0.0187 (12)
O7	0.0717 (19)	0.0268 (14)	0.0524 (16)	−0.0034 (14)	0.0168 (13)	0.0001 (13)
O8	0.0684 (17)	0.0436 (16)	0.0298 (12)	−0.0007 (14)	0.0225 (12)	−0.0067 (11)
O9	0.0775 (19)	0.0295 (13)	0.0292 (12)	0.0062 (13)	0.0000 (12)	0.0058 (10)
O10	0.0591 (16)	0.0204 (12)	0.0299 (12)	0.0018 (11)	0.0070 (11)	0.0016 (9)
O11	0.0660 (16)	0.0427 (15)	0.0298 (12)	0.0072 (13)	0.0229 (12)	−0.0033 (11)
O12	0.080 (2)	0.0350 (14)	0.0269 (12)	0.0154 (14)	0.0007 (12)	0.0059 (11)
O13	0.079 (2)	0.0174 (13)	0.0575 (16)	0.0002 (13)	0.0135 (14)	−0.0002 (12)
O14	0.081 (2)	0.0376 (15)	0.0368 (13)	0.0005 (15)	0.0155 (13)	0.0172 (12)
O15	0.0706 (19)	0.0514 (18)	0.0320 (13)	−0.0160 (15)	−0.0078 (12)	−0.0063 (12)
O16	0.112 (3)	0.0364 (17)	0.0446 (16)	0.0147 (18)	0.0118 (17)	−0.0133 (13)
N1	0.0395 (15)	0.0194 (14)	0.0404 (16)	−0.0002 (12)	0.0017 (12)	0.0029 (11)
N2	0.0445 (17)	0.0244 (15)	0.0380 (16)	−0.0015 (14)	0.0019 (13)	−0.0004 (13)
N3	0.082 (3)	0.0247 (17)	0.058 (2)	−0.0058 (18)	0.0021 (18)	−0.0120 (15)
N4	0.0573 (19)	0.0330 (18)	0.0281 (14)	−0.0092 (15)	0.0158 (13)	−0.0041 (12)
N5	0.0356 (15)	0.0281 (16)	0.0416 (16)	−0.0027 (13)	0.0110 (12)	0.0070 (13)
N6	0.0444 (16)	0.0279 (15)	0.0219 (13)	−0.0078 (13)	0.0066 (11)	−0.0031 (11)
N7	0.0428 (16)	0.0261 (15)	0.0220 (13)	−0.0012 (13)	0.0056 (11)	−0.0016 (11)
N8	0.0286 (14)	0.0255 (15)	0.0399 (15)	−0.0017 (12)	0.0096 (11)	0.0060 (12)
N9	0.0547 (18)	0.0253 (15)	0.0290 (14)	−0.0073 (14)	0.0122 (13)	−0.0055 (12)
C1	0.048 (2)	0.0270 (19)	0.0382 (19)	−0.0013 (17)	−0.0072 (16)	0.0034 (15)
C2	0.0470 (19)	0.0246 (17)	0.0274 (15)	0.0004 (15)	0.0048 (14)	0.0022 (13)
C3	0.0412 (18)	0.0329 (18)	0.0341 (17)	0.0030 (16)	0.0097 (14)	0.0045 (14)
C4	0.0338 (16)	0.0230 (16)	0.0377 (17)	−0.0032 (14)	0.0037 (13)	0.0027 (14)
C5	0.058 (2)	0.038 (2)	0.043 (2)	−0.0062 (19)	0.0018 (18)	−0.0087 (18)
C6	0.064 (3)	0.031 (2)	0.045 (2)	−0.0083 (19)	0.0001 (19)	0.0019 (18)
C7	0.0269 (15)	0.0258 (16)	0.0221 (14)	−0.0025 (13)	0.0046 (11)	−0.0039 (12)
C8	0.0311 (16)	0.0290 (17)	0.0248 (15)	−0.0051 (14)	0.0081 (12)	−0.0064 (13)
C9	0.0295 (15)	0.0314 (18)	0.0224 (14)	−0.0003 (14)	0.0090 (12)	0.0034 (13)
C10	0.0251 (14)	0.0234 (16)	0.0313 (15)	−0.0005 (13)	0.0088 (11)	0.0038 (12)
C11	0.0254 (14)	0.0262 (16)	0.0263 (14)	−0.0033 (13)	0.0082 (11)	−0.0041 (12)
C12	0.0288 (15)	0.0187 (14)	0.0213 (14)	−0.0009 (12)	0.0051 (11)	−0.0007 (11)
C13	0.0275 (15)	0.0248 (16)	0.0251 (15)	0.0014 (13)	0.0070 (12)	−0.0005 (13)
C14	0.0267 (14)	0.0246 (16)	0.0176 (14)	0.0022 (13)	0.0057 (11)	0.0006 (11)
C15	0.0256 (14)	0.0233 (15)	0.0239 (14)	0.0018 (13)	0.0057 (11)	−0.0023 (12)
C16	0.0229 (14)	0.0202 (15)	0.0276 (15)	0.0015 (12)	0.0072 (11)	0.0048 (12)
C17	0.0254 (14)	0.0274 (17)	0.0215 (14)	−0.0005 (13)	0.0074 (11)	0.0027 (12)
C18	0.0270 (15)	0.0252 (16)	0.0215 (15)	−0.0004 (13)	0.0058 (12)	−0.0030 (11)
O17W	0.126 (3)	0.0397 (18)	0.0615 (19)	−0.0171 (19)	0.043 (2)	−0.0111 (15)
O18W	0.125 (3)	0.043 (2)	0.085 (3)	0.009 (2)	0.060 (2)	0.0001 (18)

### Geometric parameters (Å, º)

**Table:**

O1—C1	1.199 (5)	N8—C16	1.448 (4)
O2—C1	1.311 (5)	N9—C18	1.468 (4)
O2—H2	0.8200	C1—C2	1.534 (5)
O3—C7	1.261 (4)	C2—C3	1.537 (5)
O4—N4	1.220 (4)	C2—H2A	1.01 (4)
O5—N4	1.220 (4)	C3—C4	1.491 (5)
O6—N5	1.232 (4)	C3—H3A	0.9700
O7—N5	1.242 (4)	C3—H3B	0.9700
O8—N6	1.230 (4)	C4—C6	1.357 (5)
O9—N6	1.224 (4)	C5—H5	0.96 (5)
O10—C13	1.249 (4)	C6—H6	0.93 (5)
O11—N7	1.225 (3)	C7—C12	1.444 (4)
O12—N7	1.224 (4)	C7—C8	1.445 (4)
O13—N8	1.222 (4)	C8—C9	1.363 (5)
O14—N8	1.230 (4)	C9—C10	1.383 (4)
O15—N9	1.228 (4)	C9—H9	0.9300
O16—N9	1.224 (4)	C10—C11	1.387 (4)
N1—C2	1.494 (4)	C11—C12	1.372 (4)
N1—H1A	0.8900	C11—H11	0.9300
N1—H1B	0.8900	C13—C14	1.451 (4)
N1—H1C	0.8900	C13—C18	1.450 (4)
N2—C5	1.328 (5)	C14—C15	1.373 (4)
N2—C4	1.380 (4)	C15—C16	1.385 (4)
N2—H2B	0.91 (5)	C15—H15	0.9300
N3—C5	1.303 (5)	C16—C17	1.388 (4)
N3—C6	1.365 (5)	C17—C18	1.361 (4)
N3—H3	0.8600	C17—H17	0.9300
N4—C8	1.463 (4)	O17W—H17A	0.84 (2)
N5—C10	1.438 (4)	O17W—H17B	0.84 (2)
N6—C12	1.461 (4)	O18W—H18A	0.83 (2)
N7—C14	1.457 (4)	O18W—H18B	0.83 (2)
			
C1—O2—H2	109.5	C6—C4—N2	105.8 (3)
C2—N1—H1A	109.5	C6—C4—C3	130.7 (3)
C2—N1—H1B	109.5	N2—C4—C3	123.5 (3)
H1A—N1—H1B	109.5	N3—C5—N2	108.3 (4)
C2—N1—H1C	109.5	N3—C5—H5	124 (3)
H1A—N1—H1C	109.5	N2—C5—H5	128 (3)
H1B—N1—H1C	109.5	C4—C6—N3	107.0 (4)
C5—N2—C4	109.0 (3)	C4—C6—H6	134 (3)
C5—N2—H2B	129 (3)	N3—C6—H6	119 (3)
C4—N2—H2B	121 (3)	O3—C7—C12	123.9 (3)
C5—N3—C6	109.8 (3)	O3—C7—C8	124.7 (3)
C5—N3—H3	125.1	C12—C7—C8	111.3 (3)
C6—N3—H3	125.1	C9—C8—C7	125.1 (3)
O5—N4—O4	123.7 (3)	C9—C8—N4	116.1 (3)
O5—N4—C8	118.8 (3)	C7—C8—N4	118.7 (3)
O4—N4—C8	117.5 (3)	C8—C9—C10	119.1 (3)
O6—N5—O7	121.8 (3)	C8—C9—H9	120.4
O7—N5—O7	0.0 (3)	C10—C9—H9	120.4
O6—N5—C10	118.9 (3)	C9—C10—C11	120.7 (3)
O7—N5—C10	119.3 (3)	C9—C10—N5	119.7 (3)
O9—N6—O8	123.9 (3)	C11—C10—N5	119.6 (3)
O9—N6—C12	119.2 (3)	C12—C11—C10	119.3 (3)
O8—N6—C12	116.9 (3)	C12—C11—H11	120.3
O12—N7—O11	122.8 (3)	C10—C11—H11	120.3
O12—N7—C14	120.2 (3)	C11—C12—C7	124.5 (3)
O11—N7—C14	117.0 (3)	C11—C12—N6	116.0 (3)
O13—N8—O14	123.4 (3)	C7—C12—N6	119.4 (3)
O13—N8—C16	119.0 (3)	O10—C13—C14	123.9 (3)
O14—N8—C16	117.6 (3)	O10—C13—C18	125.4 (3)
O16—N9—O15	123.6 (3)	C14—C13—C18	110.6 (3)
O16—N9—C18	119.5 (3)	C15—C14—C13	125.0 (2)
O15—N9—C18	116.9 (3)	C15—C14—N7	116.1 (3)
O1—C1—O2	126.3 (4)	C13—C14—N7	118.8 (3)
O1—C1—C2	121.9 (3)	C14—C15—C16	118.7 (3)
O2—C1—C2	111.7 (3)	C14—C15—H15	120.6
N1—C2—C1	107.1 (3)	C16—C15—H15	120.6
N1—C2—C3	112.3 (3)	C15—C16—C17	121.1 (3)
C1—C2—C3	115.1 (3)	C15—C16—N8	119.1 (3)
N1—C2—H2A	105 (2)	C17—C16—N8	119.8 (3)
C1—C2—H2A	111 (2)	C18—C17—C16	118.8 (3)
C3—C2—H2A	106 (2)	C18—C17—H17	120.6
C4—C3—C2	115.9 (3)	C16—C17—H17	120.6
C4—C3—H3A	108.3	C17—C18—C13	125.4 (3)
C2—C3—H3A	108.3	C17—C18—N9	117.1 (2)
C4—C3—H3B	108.3	C13—C18—N9	117.5 (3)
C2—C3—H3B	108.3	H17A—O17W—H17B	106 (3)
H3A—C3—H3B	107.4	H18A—O18W—H18B	109 (3)
			
O1—C1—C2—N1	17.5 (5)	C8—C7—C12—C11	−0.3 (4)
O2—C1—C2—N1	−164.4 (3)	O3—C7—C12—N6	−1.7 (5)
O1—C1—C2—C3	143.1 (4)	C8—C7—C12—N6	−177.6 (3)
O2—C1—C2—C3	−38.8 (4)	O9—N6—C12—C11	148.0 (3)
N1—C2—C3—C4	62.2 (4)	O8—N6—C12—C11	−30.3 (4)
C1—C2—C3—C4	−60.7 (4)	O9—N6—C12—C7	−34.5 (4)
C5—N2—C4—C6	0.3 (4)	O8—N6—C12—C7	147.2 (3)
C5—N2—C4—C3	−178.1 (3)	O10—C13—C14—C15	178.2 (3)
C2—C3—C4—C6	92.6 (5)	C18—C13—C14—C15	−5.6 (4)
C2—C3—C4—N2	−89.5 (4)	O10—C13—C14—N7	−5.3 (5)
C6—N3—C5—N2	1.7 (5)	C18—C13—C14—N7	170.9 (2)
C4—N2—C5—N3	−1.2 (5)	O12—N7—C14—C15	−150.2 (3)
N2—C4—C6—N3	0.7 (4)	O11—N7—C14—C15	28.7 (4)
C3—C4—C6—N3	179.0 (4)	O12—N7—C14—C13	33.0 (4)
C5—N3—C6—C4	−1.5 (5)	O11—N7—C14—C13	−148.1 (3)
O3—C7—C8—C9	−175.4 (3)	C13—C14—C15—C16	1.0 (5)
C12—C7—C8—C9	0.4 (4)	N7—C14—C15—C16	−175.5 (2)
O3—C7—C8—N4	2.6 (5)	C14—C15—C16—C17	4.9 (4)
C12—C7—C8—N4	178.4 (3)	C14—C15—C16—N8	−178.0 (3)
O5—N4—C8—C9	−139.3 (3)	O13—N8—C16—C15	−3.4 (4)
O4—N4—C8—C9	40.2 (4)	O14—N8—C16—C15	175.9 (3)
O5—N4—C8—C7	42.6 (5)	O13—N8—C16—C17	173.8 (3)
O4—N4—C8—C7	−138.0 (3)	O14—N8—C16—C17	−6.9 (4)
C7—C8—C9—C10	−1.4 (5)	C15—C16—C17—C18	−5.4 (4)
N4—C8—C9—C10	−179.4 (3)	N8—C16—C17—C18	177.5 (3)
C8—C9—C10—C11	2.2 (4)	C16—C17—C18—C13	0.1 (5)
C8—C9—C10—N5	179.8 (3)	C16—C17—C18—N9	176.9 (3)
O6—N5—C10—C9	3.9 (4)	O10—C13—C18—C17	−178.8 (3)
O7—N5—C10—C9	−175.1 (3)	C14—C13—C18—C17	5.1 (4)
O6—N5—C10—C11	−178.5 (3)	O10—C13—C18—N9	4.4 (5)
O7—N5—C10—C11	2.5 (4)	C14—C13—C18—N9	−171.8 (3)
C9—C10—C11—C12	−2.2 (4)	O16—N9—C18—C17	147.2 (3)
N5—C10—C11—C12	−179.7 (3)	O15—N9—C18—C17	−31.3 (4)
C10—C11—C12—C7	1.2 (5)	O16—N9—C18—C13	−35.7 (4)
C10—C11—C12—N6	178.6 (2)	O15—N9—C18—C13	145.8 (3)
O3—C7—C12—C11	175.6 (3)		

### Hydrogen-bond geometry (Å, º)

**Table:**

*D*—H···*A*	*D*—H	H···*A*	*D*···*A*	*D*—H···*A*
N1—H1*A*···O10	0.89	2.19	2.909 (3)	138
N1—H1*A*···O12	0.89	2.11	2.841 (4)	139
N1—H1*B*···O18*W*	0.89	1.85	2.700 (5)	158
N1—H1*C*···O15^i^	0.89	2.16	3.007 (4)	159
N2—H2*B*···O10	0.91 (5)	1.86 (5)	2.709 (4)	154 (4)
N2—H2*B*···O16	0.91 (5)	2.49 (4)	3.125 (4)	128 (3)
N3—H3···O9^ii^	0.86	2.56	2.992 (5)	112
N3—H3···O14^ii^	0.86	2.25	3.077 (4)	160
O2—H2···O3^iii^	0.82	1.86	2.657 (3)	165
O17*W*—H17*A*···O5^iv^	0.84 (2)	2.27 (3)	3.082 (4)	162 (9)
O17*W*—H17*B*···O3	0.84 (2)	2.14 (8)	2.864 (4)	144 (12)
O18*W*—H18*A*···O17*W*^v^	0.83 (2)	1.83 (2)	2.664 (5)	176 (5)
O18*W*—H18*B*···O7	0.83 (2)	2.32 (4)	3.005 (5)	140 (6)
C3—H3*B*···O10	0.97	2.59	3.210 (4)	122
C9—H9···O8^vi^	0.93	2.40	3.177 (4)	141
C17—H17···O11^iv^	0.93	2.36	3.177 (3)	147

Symmetry codes: (i) *x*+1, *y*, *z*+1; (ii) −*x*+1, *y*−1/2, −*z*+1; (iii) −*x*+2, *y*−1/2, −*z*+2; (iv) *x*, *y*, *z*−1; (v) −*x*+2, *y*−1/2, −*z*+1; (vi) *x*, *y*, *z*+1.
